# Retention of Mandibular Complete Overdentures using Mini Dental Implants (Ø < 3 mm) and Standard Diameter Implants (Ø > 3mm): A Systematic Review and Meta-Analysis of Randomised Controlled Trials

**DOI:** 10.3290/j.ohpd.b5282167

**Published:** 2024-05-07

**Authors:** Suha Mohammed Aljudaibi, Mohammad Abdullah Zayed Alqhtani, Asmaa Saleh Almeslet, Omir Aldowah, Khalid Dhafer S. Alhendi

**Affiliations:** a Assistant Professor, Department of Preventive Dental Sciences, College of Dentistry, Princess Nourah bint Abdulrahman University, P.O. Box 84428, Riyadh 11671, Saudi Arabia. Designed the study, wrote the manuscript.; b Assistant Professor, Prosthetic Dental Science Department, Faculty of Dentistry, Najran University, Najran, Saudi Arabia. Wrote the methods and discussion.; c Assistant Professor, Department of Oral Maxillofacial Surgery and Diagnostic Sciences, College of Medicine and Dentistry, Riyadh Elm University, Riyadh, Saudi Arabia. Wrote the methods and discussion.; d Associate Professor, Prosthetic Dental Science Department, Faculty of Dentistry, Najran University, Najran, Saudi Arabia. Performed the literature search.; e Assistant Professor, Prosthetic Dental Science Department, Faculty of Dentistry, Najran University, Najran, Saudi Arabia. Performed the literature search, wrote the discussion.; All authors contributed equally and read and revised the manuscript before submission.

**Keywords:** edentulous, implant survival rate, mandible, mini dental implants, overdenture, standard-diameter implants

## Abstract

**Purpose:**

The objective of the present systematic review and meta-analysis was to assess randomised controlled trials (RCTs) which assessed the efficacy of mini dental implants (MDIs) and standard-diameter implants (SDIs) in retaining mandibular overdentures (MO).

**Materials and Methods:**

The focused question was “Is there a difference in the mechanical stability between MDIs and SDIs in retaining MO?” Indexed databases were searched up to and including November 2023 using different keywords. Boolean operators were used during the search. The literature was searched in accordance with the PRISMA guidelines. The PICO characteristics were: patients (P) = individuals with complete mandibular dentures requiring dental implants; Intervention (I) = placement of MDIs under mandibular dentures; Control (C) = placement of SDIs under mandibular dentures; Outcome (O) = comparison of stability between MDIs and SDIs in supporting mandibular dentures. Only RCTs were included. Risk of bias (RoB) was assessed using the Cochrane RoB tool.

**Results:**

Five RCTs were included. The numbers of participants ranged between 45 and 120 edentulous individuals wearing complete mandibular dentures. The mean age of patients ranged between 59.5 ± 8.5 and 68.3 ± 8.5 years. The number of MDIs and SDIs ranged between 22 and 152 and 10 and 80 implants, respectively. The follow-up duration ranged between one week and 12 months. Three RCTs reported an improvement in the quality of life (QoL) of all patients after stabilisation of mandibular dentures using MDIs or SDIs. In one RCT, peri-implant soft tissue profiles were comparable between MDIs and SDIs at the 1-year follow-up. The implant survival rate was reported in two RCTs, which were from 89% to 98% and 99% to 100% for MDIs and SDIs, respectively. All RCTs had a low RoB.

**Conclusion:**

Mini dental implants represent a viable alternative to traditional standard-diameter implants when seeking optimal retention for mandibular overdentures.

Acclimating to dentures poses a notable challenge, as wearers may experience issues such as a heightened gag reflex and compromised taste perception.^25,30^ Moreover, in patients with thin or resorbed alveolar ridges, achieving denture stabilisation during mastication and communication presents additional challenges.^1^ Particularly, the stability of mandibular dentures becomes a significant concern due to the inherent challenges associated with jaw and tongue movements, leading to frequent denture destabilisation.^1^ Addressing such challenges traditionally involves the use of denture adhesive pastes, a practice with its own set of inconveniences. The adhesive paste often exacerbates the problem by adhering to the alveolar ridges, palate and fitting surfaces of dentures, which often complicates the removal process, posing an inconvenience for patients.^2^

Dental implants possess the potential to fuse with surrounding bone (osseointegration) and can remain esthetically and functionally stable in partially and/or completely edentulous individuals.^13,22^ Mini dental implants (MDIs), characterised by their reduced dimensions (diameters and lengths ranging between 1.8 and <3.0 and 9 and 15 mm, respectively) and one-piece structure, have emerged as a noteworthy innovation in contemporary implant dentistry.^24,28^ The advent of MDIs represents a paradigm shift in prosthodontics, offering a minimally invasive solution for enhanced denture stabilisation and implant-supported restorations. In the field of prosthetic dentistry, MDIs have gained attention for their potential role in improving denture stabilisation, especially in cases where traditional standard-diameter implants (SDIs) with diameters ≥ 3 mm might be challenging to place due to limited space and/or anatomical considerations.^4,11,24^ With regard to replacement of a single missing tooth, Roccuzzo et al^21^ reported that MDIs are as reliable as Ø3.3-mm implants, in terms of technical and biological complications as well as changes in crestal bone levels. It is also worth mentioning that MDIs serve as a viable alternative to SDIs in narrow alveolar ridges.^9^ Additionally, the insertion procedures are more straightforward and less time consuming, employing a reduced set of drills and often utilising a flapless approach.^7,17,20,31,32^ From a financial perspective, MDIs present economic appeal as well, given their lower cost in comparison to standard fixtures and the reduced operative time required.^8,29^ In a randomised controlled trial (RCT) with a 60-month follow-up, Celebic et al^5^ evaluated the clinical outcome of three vs four MDIs used for retention of mandibular overdentures (MO). The survival rates of MDIs were evaluated at follow-up. The results showed that the survival rates of three and four MDIs were approximately 94% and 92%, respectively. The RCT concluded that in patients with narrow alveolar ridges, insertion of three MDIs is as successful as using four MDIs for retention of MO.^5^ Although this study acknowledges the efficacy of MDI for supporting MO, Celebic et al^5^ did not compare MDIs and SDIs.^5^ In another RCT, Zygogiannis et al^31^ assessed the peri-implant modified plaque index (mPI), sulcular bleeding index (SBI), peri-implant probing depth (PD) and crestal bone levels (CBL) around SDIs and MDIs placed under MO at baseline and after 12 months. The results showed MDIs and SDIs are suitable fixtures for retaining MO, and the soft tissue profiles and peri-implant CBL of MDIs are similar to those of SDIs.^31^ Nevertheless, it has been suggested that despite being classified as dental implants, MDIs demonstrate unique behaviour when subjected to functional loads.^10^ Therefore, operator discretion should be exercised when using MDIs in clinical practice.^10^ A careful review of indexed literature showed that, to date, no studies exist that have systemically reviewed RCTs which assessed the efficacy of MDIs and SDIs in retaining MO.

With this background, the objective of the present systematic review and meta-analysis was to assess randomised controlled trials that assessed the efficacy of MDIs and SDIs in retaining MO.

## Materials and Methods

### Focused Question

The focused question was “Is there a difference in the mechanical stability between MDIs and SDIs in retaining MO?”

### Population, Intervention, Control, Outcome (PICO) Approach

To enhance the effectiveness of the literature search, study selection, and analyses, the Population/Patients, Intervention, Control, Outcome (PICO) approach was employed in the following manner: Patients (P) = individuals with mandibular complete dentures requiring dental implants; Intervention (I) = placement of MDIs under mandibular dentures; Control (C) = placement of SDIs under mandibular dentures; Outcome (O) = comparison of MS between MDIs and SDIs in supporting mandibular dentures.

### Eligibility Criteria

Only RCTs were considered eligible for inclusion in the present systematic review. Letters to the Editor, case reports, in-vitro studies, case series, observational studies, epidemiological investigations, studies on animal models, commentaries and expert opinions/perspectives were excluded.

### Literature Search

The present evidence-based review was performed in accordance with the Preferred Reporting Items for Systematic Reviews and Meta-Analyses (PRISMA) guidelines^19^ to minimise bias, enhance the reproducibility of the methods, and provide a clear and structured account of the review process.

### Search Strategy and Study Selection

An electronic search of indexed databases (PubMed, Web of Knowledge, Scopus, and Ovid) and Google Scholar was performed up to and including November 2023 without language or time restrictions. The following MESH terms were used in different combinations: (1) mini implants, (2) mini dental implants, (3) implant survival rate, (4) implant success rate (5) implant failure rate, (6) complications, (7) denture, and (8) overdenture. Boolean operators (OR, AND) were used in conjunction with these keywords to expand search results. Search results were screened based on the above-mentioned protocol by title and abstract, and full texts of relevant studies were reviewed independently by one author (SA). Disagreements were resolved through discussion and consultation with a second author (MA). Manual searching of the reference lists of pertinent original and review articles was also conducted to identify relevant studies that may have been missed in the previous search strategy. The pattern of the present study was customised to primarily summarise the pertinent information.

### Risk of Bias Assessment

The Cochrane Risk of Bias (RoB) tool^14^ was applied to evaluate bias across key domains, including random sequence generation, allocation concealment, blinding of participants and personnel, blinding of outcome assessment, handling of incomplete outcome data, selective reporting, and other potential sources of bias. Ratings of “low”, “unclear”, or “high” RoB were assigned to each domain based on the evaluation. The overall risk of bias for each study was then determined by summarising individual domain ratings.

### Meta-Analysis

A meta-analysis was conducted utilising Review Manager (RevMan), version 5.2 (The Nordic Cochrane Centre, The Cochrane Collaboration, 2012). Means and standard deviations were computed and consolidated from the selected articles. Each study provided the requisite data, including mean, standard deviation, and sample size, facilitating the calculation of effect size via Cohen’s d and its corresponding standard error. Leveraging Cohen’s d and its standard error, odds ratios were derived employing the Logit method, along with confidence intervals encompassing lower and upper limits.

## Results

### General Characteristics of Included RCTs

**Fig 1:**
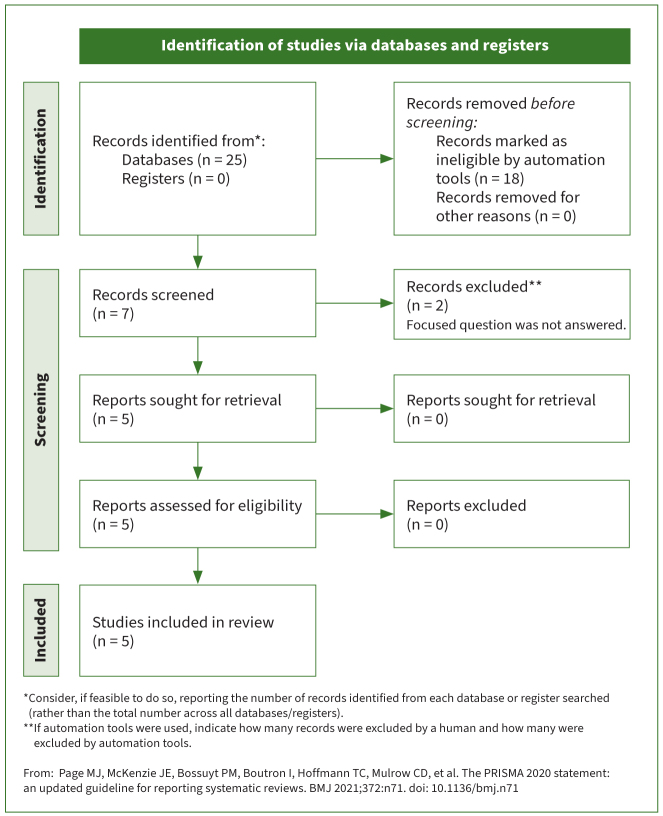
PRISMA flowchart.

The initial search yielded 25 studies. Twenty studies which did not fulfill the eligibility criteria and/or failed to stringently abide by the PICO standards were excluded (Appendix A). In total, 5 RCTs^7,17,20,31,32^ were included and processed for data extraction (Fig 1). The study by Jawad et al^17^ was a pilot RCT. The numbers of participants ranged between 45 and 120 edentulous individuals wearing mandibular complete dentures. The mean ages of patients ranged between 59.5 ± 8.5 and 68.3 ± 8.5 years. The numbers of males and females ranged between 19 and 39 and 26 and 81 individuals, respectively. The number of MDIs and SDIs ranged between 22 and 152 and 10 and 80 implants, respectively. The follow-up duration ranged from one week to 12 months in the RCTs.^7,17,20,31,32^ Prior sample size estimation (SSE) was done in three^7,20,31^ of the five RCTs^7,17,20,31,32^ (Table 1). In all RCTs,^7,17,20,31,32^ MDIs and SDIs were placed in the interforamina region of the mandible.

**Table 1:** General characteristics of included randomised controlled trials

Authors	Participants (n)	Mean age (range)	Gender	Groups (no. of implants)	Follow-up	Evaluations at follow-up	Power analysis
de Souza et al^7^	120	59.5 ± 8.5 years	39 males 81 females	Test 1: 4 MDIs (n = 152) Test 2: 2 MDIs (n = 84) Control: 2 SDIs (n = 80)	1 year	QoL Pain Chewing ability Implant survival rate	Yes
Jawad et al^17^	45 patients	68.3 ± 8.5 years	19 males 26 females	Test: MDIs (n = 22) Control: SDIs (n = 23)	6 months	QoL Self-rated pain	No
Ribeiro et al^20^	120	59.5 ± 8.5 years	39 males 81 females	Test 1: 4 MDIs (n = 152) Test 2: 2 MDIs (n = 84) Control: 2 SDIs (n = 80)	One week	Pain Discomfort Chewing ability	Yes
Zygogiannis et al^31^	50 patients	67.9 ± 7.7 years	24 males 26 females	Test: 4 MDIs (n = 25) Control 1: 2 immediately loaded SDIs (n = 15) Control 2: 2 delayed-loaded SDIs (n = 10)	1 year	QoL	No
Zygogiannis et al^32^	50 patients	67.9 ± 7.7 years	24 males 26 females	Test: 4 MDIs (n = 25) Control 1: 2 immediately loaded SDIs (n = 15) Control 2: 2 delayed-loaded SDIs (n = 10)	1 year	PD CBL mPI SBI	Yes

CBL: crestal bone loss; SDIs: standard diameter implants; MDIs: mini dental implants; mPI: modified plaque index; PD: Probing depth; QoL: quality of life; SBI: sulcular bleeding index.

### Implant-related Characteristics

In all RCTs,^7,17,20,31,32^ the MDIs were placed transmucosally, whereas the SDIs were placed after reflection of surgical flaps. As a preoperative measure in three studies,^17,31,32^ patients were instructed to rinse with 0.2% CHX for 60 s, and in three other studies,^7,17,20^ patients were administered 2 g of amoxicillin orally as a preoperative measure. The numbers of MDIs and SDI ranged between 2 and 316 and 2 and 80 fixtures, respectively.^7,17,20,31,32^ The diameters and lengths of MDIs ranged from 1.8 to 2.4 and 10 to 18 mm, respectively.^7,17,20,31,32^ The diameters and lengths of SDIs ranged from 3 to 4.1 and 10 to 12 mm, respectively.^7,17,20,31,32^ In four RCTs,^7,17,31,32^ the MDIs and SDIs were inserted at an insertion torque of 35 Ncm. The study by Ribeiro et al^20^ did not report the implant insertion torque. Drilling speed of the pilot drill was not reported in any of the RCTs.^7,17,20,31,32^ MDIs were immediately loaded in two RCTs,^31,32^ wherease in the study by de Souza et al,^7^ delayed loading of MDIs and SDIs was performed. In the studies by Jawad et al^17^ and Ribeiro et al,^20^ early loading of MDIs and SDIs was performed (Table 2).

**Table 2:** Implant-related characteristics

Authors	Preoperative management	Mini dental implants		Conventional dental implants		Drilling speed	Insertion torque	Implant loading
		Total implants	D x L	Total implants	D x L			
de Souza et al^7^	Oral dose of 2 g amoxicillin	316*	2 x 10 mm	80†	4 x 10 mm	NR	NR	MDIs: DL SDIs: DL
Jawad et al^17^	Oral dose of 2 g amoxicillin or 600 mg clindamycin Oral rinse with 0.2% CHX for 60 s	2*	2.1 x 10 mm	2†	3 x 11 mm	NR	MDIs: 35 Ncm SDIs: 35 Ncm	After 60 days (early)
Ribeiro et al^20^	Oral dose of 2 g amoxicillin	316*	2 x 10 mm	80†	4 x 10 mm	NR	NR	Test 1 and 2: after 1 week (early) Control: DL
Zygogiannis et al^31^	Oral rinse with 0.2% CHX for 60 s	100*	1.8 or 2.1 or 2.4 x 10–18 mm	50†	3.3 or 4.1 mm x 10 or 12 mm	NR	MDIs: 35 Ncm SDIs: 35 Ncm	IL in MDIs Control 1: IL Control 2: DL
Zygogiannis et al^32^	Oral rinse with 0.2% CHX for 60 s	100*	1.8 or 2.1 or 2.4 x 10–18 mm	50†	3.3 or 4.1 mm x 10 or 12 mm	NR	MDIs: 35 Ncm SDIs: 35 Ncm	IL in MDIs Control-1: IL Control-2: DL

*Placed transmucosally (flapless); †placed after flap elevation; SDIs: standard diameter implants; MDIs: mini dental implants DL: delayed loading; IL: immediate loading.

### Outcomes

Three RCTs^17,20,32^ reported an improvement in the quality of life (QoL) of all patients after stabilisation of mandibular dentures using MDIs or SDIs. In these RCTs,^17,20,32^ there was no statistically significant difference in the QoL among patients who received MDIs or SDIs. In the RCT by Ribeiro et al,^20^ self-rated pain scores after implant placement were higher with MDIs than SDIs. This self-rated pain was assessed one week after placement of MDIs. In the remaining RCTs, there was no statistically significant difference in self-rated pain and discomfort between MDIs and SDIs. In the study by Zygogiannis et al,^31^ peri-implant soft tissue profiles (mPI, PD, SBI and CBL) were statistically comparable between MDIs and SDIs at the 1-year follow-up. The implant survival rate was reported in two RCTs,^7,31^ ranging from 89% to 98% and 99% to 100% for MDIs and SDIs, respectively (Table 3).

**Table 3:** Outcomes of studies

Authors	Analgesic intake	Self-rated mucosal pain	QoL	Soft tissue complications	Implant failures (n)	Implant survival rate
de Souza et al^7^	NR	No significant difference between MDIs and SDIs	Superior in patients treated with MDIs than SDIs.	NR	Test 1: 10 Test 2: 9 Control: 0	Tests 1 and 2: 89% Control: 99%
Jawad et al^17^	Higher in SDI group*	None	Improved in both groups compared with baseline with no difference between MDIs and SDIs.	None reported	None	NR
Ribeiro et al^20^	NR	Higher in Test 1 compared with other groups†	Improved in all groups compared with baseline with no difference between MDIs and SDIs.	NR	NR	NR
Zygogiannis et al^31^	NR	No difference between MDIs and SDIs†	Improved in all groups compared with baseline with no difference between MDIs and SDIs.	NR	NR	NR
Zygogiannis et al^32^	NR	Similar between MDIs (36%) and SDIs (40%)†	NR	No difference in mPI, SBI, PPD and CBL in all groups	Test: 2 Control 1: 0 Control 2: 4	Group 1: 98% Group 2: 100% Group 3: 100%

*Compared with MDIs; †pain was assessed during initial stages of the study (after implant placement).

### Risk of Bias Assessment

**Fig 2:**
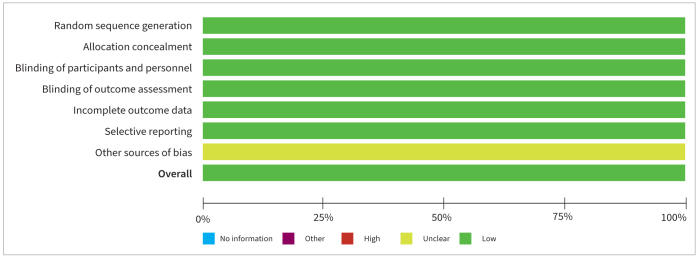
Risk of bias assessment.

All RCTs^7,17,20,31,32^ had a low RoB, as shown in Fig 2.

### Meta-Analysis

**Fig 3:**
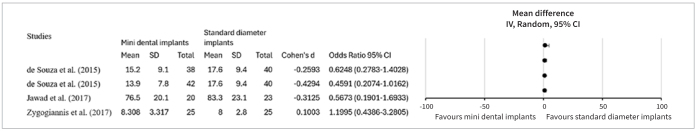
Quantitative evaluation (meta0-analysis) of included studies.

A meta-analysis was performed a using random effects model due to high heterogeneity (I^2^=80%) among the studies eligible for quantitative analysis. A meta-analysis was done on three studies.^7,17,32^ The study by de Souza et al^7^ had two treatment groups with a control, and the present authors calculated it as a separate study for meta-analysis. The overall effect, reported in forest plot (Fig 3), revealed that there was no difference in oral-health quality of life among patients wearing complete mandibular overdentures retained by MDIs and SDIs.

## Discussion

The consensus of the present systematic review and meta-analysis is that MDIs are a suitable replacement for SDIs for achieving retention of MO. In the current systematic review, the authors meticulously applied stringent PICO criteria, ensuring a judicious selection of studies for inclusion. Notably, the focus was exclusively on RCTs,^7,17,20,31,32^ recognised as the epitome of research methodology due to their exacting design, which minimises biases and facilitates the establishment of causal relationships between interventions and outcomes, as emphasised by a previous study.^26^ This methodological rigor was further augmented by the deliberate inclusion of only RCTs, affording each study^7,17,20,31,32^ a built-in “control group.” In the context of the present systematic review, SDIs used for the retention of MO was considered the control group. The synthesised results from the selected studies consistently demonstrated that MDIs emerge as a reliable and effective therapeutic modality for enhancing the retention of MO. Remarkably, the survival rates of MDIs were found to be comparable to their SDI counterparts. In addition, the meta-analysis results showed that the overall effect size was not statistically significant, that is, the suitability of MDIs for retention of mandibular overdentures was not superior to that attained from SDI or vise-versa. One explanation could be the less invasive nature of the surgical protocol implemented with MDIs. MDIs are typically inserted transmucosally (flaplessly), contrasting with the more extensive approach need for SDIs, in which a soft tissue flap is surgically reflected. This distinction not only underscores the clinical advantages of MDIs but also aligns with contemporary preferences for minimally invasive procedures. The results of a histological study are noteworthy,^27^ in which MDIs exhibited the ability to undergo osseointegration without the development of fibrous tissues between the threads and the adjacent osseous tissues. While the results of the present systematic review uniformly favour MDIs as a viable alternative to traditional SDIs, it is imperative to acknowledge various factors that possibly contributed to this conclusion.

Patient selection in interventional research is critical, as it influences the robustness, generalisability, and ethical underpinnings of study outcomes. Rigorous patient selection enhances the internal validity of a study by minimising confounding variables. Homogeneity in the study population allows researchers to attribute observed effects more confidently to the intervention under investigation. Moreover, patient selection criteria impact the external validity of a study, influencing the extent to which findings can be generalised to the broader patient population. A meticulous examination of the included RCTs revealed that individuals receiving either MDIs or SDIs demonstrated systemic health, with no use of combustible or non-combustible nicotinic products. It is widely recognised that a compromised immune system, often evident in individuals with poorly-controlled diabetes mellitus, constitutes a risk factor for both periodontal and peri-implant diseases.^12,16,18^ Moreover, the literature underscores the substantial influence of the health status of peri-implant tissues on the long-term success and survival of dental implants.^23^ The authors of the present study speculate that all patients evaluated in the included RCT were cleaning implant surfaces routinely and this factor seems to have contributed towards minimising the risk of increased peri-implant mPI, PD and gingival bleeding (SBI) as reflected in the study by Zygogiannis et al.^31^ The authors of the present study speculate that habitual use of nicotinic products, a compromised immune status and poor oral-hygiene maintenance enhance the risk of peri-implant soft tissue inflammation and loss of crestal bone; this relationship is independent of implant dimensions.

The SSE or power analysis (PA) is a critical aspect of experimental design and statistical analysis in research.^3^ It is described as the probability that a study will correctly reject a false null hypothesis (i.e., avoid a Type II error).^3^ Prior SSE was performed in three^7,20,31^ of the RCTs. The study by Jawad et al^17^ was a pilot RCT, and the authors pointed to the lack of PA as a potential limitation of their RCT. Likewise, in the study by Zygogiannis et al,^32^ PA was not performed and the authors stated that even though SSE was not based on patient-based outcomes, the power of their study^32^ was deemed sufficient to detect statistically significant differences between the groups. In the opinion of the present authors, the justification for not performing power analysis is scientifically invalid. Hence, results reported in the RCT by Zygogiannis et al^32^ should be interpreted with caution. A major limitation of three of the RCTs^7,31,32^ was their relatively short follow-up duration of 12 months. Moreover, it is well acknowledged that local and systemic factors, such as habitual use of combustible nicotinic products and being immunosuppressed (e.g., patients with poorly-controlled diabetes mellitus), are at an increased risk of developing peri-implant diseases compared to non-smokers and immunocompetent individuals.^6,15,16^ It is therefore likely that such risk factors pose a threat to the stability and function of MDIs as well; however, there are no studies to date that have investigated the long-term success and survival of MDIs in tobacco-product users and patients with metabolic diseases such as diabetes. However, it is speculated that adoption of vigilant criteria in terms of patient/case selection, routine visits to oral healthcare providers and routine oral hygiene maintenance are critical for long-term peri-implant health and stability.

## Conclusion

MDIs are a feasible substitute for conventional SDIs when aiming for optimal retention of MO. It is imperative to conduct additional RCTs with extended follow-up periods, spanning a minimum of five years, to comprehensively evaluate the performance of MDIs in retaining MO as compared to SDIs.

## References

[ref1] Albuquerque IS, Regis RR, de Souza RF, Gurgel KF, Silva PG, Pinto-Fiamengui LMS (2020). Is a two-step impression mandatory for complete denture fabrication on the severely resorbed mandible? A randomized trial on patient perception and denture quality. J Dent.

[ref2] Bogucki ZA, Napadlek P, Dabrowa T (2015). A clinical evaluation denture adhesives used by patients with xerostomia. Medicine (Baltimore).

[ref3] Bolarinwa OA (2020). Sample size estimation for health and social science researchers: The principles and considerations for different study designs. Niger Postgrad Med J.

[ref4] Bulard RA, Vance JB (2005). Multi-clinic evaluation using mini-dental implants for long-term denture stabilization: a preliminary biometric evaluation. Compend Contin Educ Dent.

[ref5] Celebic A, Kovacic I, Petricevic N, Alhajj MN, Topic J, Junakovic L (2023). Clinical outcomes of three versus four mini-implants retaining mandibular overdenture: a 5-year randomized clinical trial. Medicina.

[ref6] Chackartchi T, Romanos GE, Sculean A (2019). Soft tissue-related complications and management around dental implants. Periodontol 2000.

[ref7] de Souza RF, Ribeiro AB, Della Vecchia MP, Costa L, Cunha TR, Reis AC (2015). Mini vs. standard implants for mandibular overdentures: a randomized trial. J Dent Res.

[ref8] Della Vecchia MP, Leles CR, Cunha TR, Ribeiro AB, Sorgini DB, Muglia VA (2018). Mini-implants for mandibular overdentures: cost-effectiveness analysis alongside a randomized trial. JDR Clin Trans Res.

[ref9] Duraisamy R, Ganapathy DM, Rajeshkumar S, Ashok V (2022). Mini-implants in dentistry – a review. J Long Term Eff Med Implants.

[ref10] Flanagan D (2021). Rationale for mini dental implant treatment. J Oral Implantol.

[ref11] Flanagan D, Mascolo A (2011). The mini dental implant in fixed and removable prosthetics: a review. J Oral Implantol.

[ref12] Graves DT, Ding Z, Yang Y (2020). The impact of diabetes on periodontal diseases. Periodontol 2000.

[ref13] Guglielmotti MB, Olmedo DG, Cabrini RL (2019). Research on implants and osseointegration. Periodontol 2000.

[ref14] Higgins JP, Altman DG, Gøtzsche PC, Jüni P, Moher D, Oxman AD (2011). The Cochrane Collaboration’s tool for assessing risk of bias in randomised trials. BMJ.

[ref15] Javed F, Rahman I, Romanos GE (2019). Tobacco-product usage as a risk factor for dental implants. Periodontol 2000.

[ref16] Javed F, Romanos GE (2019). Chronic hyperglycemia as a risk factor in implant therapy. Periodontol 2000.

[ref17] Jawad S, Barclay C, Whittaker W, Tickle M, Walsh T (2017). A pilot randomised controlled trial evaluating mini and conventional implant retained dentures on the function and quality of life of patients with an edentulous mandible. BMC Oral Health.

[ref18] Nibali L, Gkranias N, Mainas G, Di Pino A (2022). Periodontitis and implant complications in diabetes. Periodontol 2000.

[ref19] Page MJ, Moher D, Bossuyt PM, Boutron I, Hoffmann TC, Mulrow CD (2021). PRISMA 2020 explanation and elaboration: updated guidance and exemplars for reporting systematic reviews. BMJ (Clinical research ed).

[ref20] Ribeiro AB, Della Vecchia MP, Cunha TR, Sorgini DB, Dos Reis AC, Muglia VA (2015). Short-term post-operative pain and discomfort following insertion of mini-implants for retaining mandibular overdentures: a randomized controlled trial. J Oral Rehabil.

[ref21] Roccuzzo A, Imber JC, Lempert J, Hosseini M, Jensen SS (2022). Narrow diameter implants to replace congenital missing maxillary lateral incisors: A 1-year prospective, controlled, clinical study. Clin Oral Implants Res.

[ref22] Sailer I, Karasan D, Todorovic A, Ligoutsikou M, Pjetursson BE (2022). Prosthetic failures in dental implant therapy. Periodontol 2000.

[ref23] Schwarz F, Ramanauskaite A (2022). It is all about peri-implant tissue health. Periodontol 2000.

[ref24] Shatkin TE, Petrotto CA (2012). Mini dental implants: a retrospective analysis of 5640 implants placed over a 12-year period. Compend Contin Educ Dent.

[ref25] Steas AD (1997). Overcoming altered taste sensation in complete denture wearers. J Prosthet Dent.

[ref26] Turner L, Shamseer L, Altman DG, Weeks L, Peters J, Kober T (2012). Consolidated standards of reporting trials (CONSORT) and the completeness of reporting of randomised controlled trials (RCTs) published in medical journals. Cochrane Database Syst Rev.

[ref27] Van Doorne L, Meijer G, Cuijpers V, De Bruyn H (2022). Histomorphometric analysis of flaplessly placed and early loaded one-piece mini dental implants in overdenture patients. Int J Periodont Restor Dent.

[ref28] Vigolo P, Givani A (2000). Clinical evaluation of single-tooth mini-implant restorations: a five-year retrospective study. J Prosthet Dent.

[ref29] Vogel R, Smith-Palmer J, Valentine W (2013). Evaluating the health economic implications and cost-effectiveness of dental implants: a literature review. Int J Oral Maxillofac Implants.

[ref30] Yildirim-Bicer AZ, Akarslan ZZ (2014). Influence of gag reflex on removable prosthetic restoration tolerance according to the patient section of the short form of the Gagging Problem Assessment Questionnaire. J Adv Prosthodont.

[ref31] Zygogiannis K, Aartman IH, Parsa A, Tahmaseb A, Wismeijer D (2017). Implant mandibular overdentures retained by immediately loaded implants: a 1-year randomized trial comparing the clinical and radiographic outcomes between mini dental implants and standard-sized implants. Int J Oral Maxillofac Implants.

[ref32] Zygogiannis K, Aartman IH, Wismeijer D (2018). Implant mandibular overdentures retained by immediately loaded implants: a 1-year randomized trial comparing patient-based outcomes between mini dental implants and standard-sized implants. Int J Oral Maxillofac Implants.

